# Role of Gastroesophageal Reflux Disease in Morbidity and Mortality for Patients Admitted With Pulmonary Hypertension

**DOI:** 10.7759/cureus.39431

**Published:** 2023-05-24

**Authors:** Anmol Mittal, Afif Hossain, Daniel Wang, Ayham Khrais, Sushil Ahlawat, Keith Guevarra, Julius Gardin

**Affiliations:** 1 Department of Medicine, Rutgers University New Jersey Medical School, Newark, USA; 2 Department of Gastroenterology and Hepatology, Rutgers University New Jersey Medical School, Newark, USA; 3 Department of Pulmonary and Critical Care Medicine, Rutgers University New Jersey Medical School, Newark, USA; 4 Department of Cardiology, Rutgers University New Jersey Medical School, Newark, USA

**Keywords:** national inpatient sample (nis), phtn, proton-pump inhibitors (ppi), gerd, pulmonary hypertension

## Abstract

Introduction: The association between gastroesophageal reflux disease (GERD) and morbidity and mortality in patients with pulmonary arterial hypertension (PH) is unknown. Our objective was to examine the difference in socio-demographics, comorbidities, and morbidity/mortality in PH patients also diagnosed with GERD, compared to PH patients without GERD.

Methods: We performed a retrospective cross-sectional study of the large U.S. National Inpatient Sample identifying patients with a primary diagnosis of primary pulmonary hypertension (PH). All patients ≥ 18 years old that were admitted with a primary diagnosis of PH from January 1, 2001, to December 31, 2013, in the NIS database were included. We analyzed the socio-demographic and clinical comorbidities in PH patients with and without GERD. We investigated the predictors for complications of PH and differences in hospital utilization in this population.

Results: PH patients with GERD were more likely to be older than 18-29 years. They were more likely to be Caucasian and female and less likely to be part of the top 75% median income compared to the bottom 25%. Patients with GERD were more likely insured with Medicare or private insurance but less likely to have Medicaid or be uninsured. Patients were more likely to be obese, and have asthma, chronic bronchitis, obstructive sleep apnea, hypertension, and hypothyroidism but were less likely to have diabetes or a history of alcohol use. PH Patients with GERD were less likely to have myocardial infarctions, cardiac arrests, pulmonary embolisms, pulmonary hemorrhages, cardiac interventions, acute respiratory failure, acute renal failure, or urinary tract infections compared to those without GERD. Patients with GERD were, however, more likely to have acute heart failure exacerbations and aspiration pneumonia. Patients with a diagnosis of GERD had lower mortality, length of stay (LOS), and hospital costs compared to their counterparts.

Conclusions: The concomitant presence of GERD is associated with fewer adverse outcomes in patients with PH. Though it is well understood that treatment of GERD is beneficial for lung disease, the exact role of GERD in PH has not been identified. This study helps characterize the important role appropriately treated GERD may play in preventing morbidity and mortality due to PH.

## Introduction

Pulmonary hypertension (PH) is a condition involving high lung vasculature pressure. It commonly presents as an insidious onset of dyspnea on exertion. Untreated, this condition has a mortality rate of 32% in the first year [1]. The World Health Organization classifies five major groups for causes of PH which include pulmonary arterial hypertension, left heart disease, lung pathology, and others, the most common being left heart dysfunction. Other secondary causes and signs of PH are less well-known [2]. Among these is the association between PH and gastroesophageal reflux disease (GERD).

The pathophysiology of GERD in the development of PH is incompletely understood. One of the mechanisms postulated is the tendency for micro-aspirations to occur in patients with reflux [3]. These aspirations lead to erosion and subsequent damage to the lung parenchyma, promoting the development of interstitial lung disease. In addition, chronic aspiration triggers bronchospasm in response to the mucosal insult. This pernicious combination of irritation and lung reflex has already associated reflux with other lung conditions such as asthma [4]. It has been postulated that these same insults could increase parenchymal damage and cause lung pathology associated with PH. Furthermore, lung pathologies such as pulmonary fibrosis have been linked to PH [5].

The association between GERD and PH involves a fundamental shift in baseline gastrointestinal pH. Due to a decrease in the lower esophageal sphincter, acidic stomach contents reflux above the diaphragm into the upper thoracic and cervical esophagus. This results in a drop in pH from seven to as low as four; H+ has been hypothesized to lead to bronchial irritation and contribute to lung disease [6]. Proton pump inhibitors (PPIs) have been shown to prevent lung disease [7].

Our investigation seeks to further elucidate the association between GERD and PH as well as the role the former has on the development of cardiac and lung pathology sequelae.

This article was previously presented as a meeting abstract at the 2020 ATS Annual Scientific Meeting on May 15, 2021 [8].

## Materials and methods

Data source

The National Inpatient Sample (NIS) database was used for a retrospective analysis of inpatient admissions in the United States from January 1, 2001 through December 31, 2013. The NIS database is a publicly accessible database available as part of the Healthcare Cost and Utilization Project (HCUP), which has been validated as an effective tool to assess inpatient data. The database is inclusive of about 8 million hospitalizations every year and represents 20% of the admissions from about 1000 hospitals in the 47 continental United States and the District of Columbia, encompassing 97% of the United States population. Available in the database are key elements for analysis such as primary and associated admission diagnoses, inpatient mortality, length of stay (LOS), cost of admission, types of admission, payor source, and socio-demographics (race, median income status, i.e.). HCUP provides an estimated discharge weight which is computed to generate national estimates. All diagnoses, procedures, and comorbidities in the NIS database are coded using the International Classification of Diseases, Ninth Revision (ICD-9) codes. As this is a publicly available database that is de-identified for use, there is no Institutional Review Board approval required for this study, and informed consent is waived.

Study population and variables

Data of patients aged 18 years old or older, at the time of admission with a primary discharge diagnosis of primary PH, were extracted using ICD-9 diagnosis code 416.0 (Table 1). The ICD-9 coding differentiates between the World Health Organization (WHO) category I and categories II-V through the use of two ICD codes, 416.0 and 416.8 respectively. In this study, the selective use of primary pulmonary arterial hypertension was made using ICD code 416.0. Therefore, throughout the article, PH refers strictly to primary pulmonary hypertension.

**Table 1:** ICD-9 diagnostic and procedure codes used for the study ICD-9: International Classification of Diseases, Ninth Revision

ICD 9 diagnostic code	Diagnosis
416.0	Pulmonary hypertension
530.81	Gastroesophageal reflux disease
493	Asthma
490, 492, 494, 496	Chronic bronchitis
327.73	Obstructive sleep apnea
507	Aspiration pneumonia
787	Dysphagia
786.3X	Pulmonary hemorrhage
415	Pulmonary embolism
416.9, 415.0	Right heart failure, cor pulmonale
410	Myocardial infarction
433.X1, 434.X1	Ischemic stroke
427.5	Cardiac arrest
428.X1, 428.X3	Acute heart failure exacerbation
518.81	Acute respiratory failure
584	Acute renal failure
599.0, 997.5	Urinary tract infection
291.X, 291.8X, 535.30, 535.31, 305.0X, 357.5, 425.5, 303.0X, 303.9X 571.X	Alcohol use
305.1, V158.1	Smoking history, tobacco use
ICD 9 Procedure code	Procedure description
88.5X, 37.2X	Diagnostic cardiac testing

All patient socio-demographics were obtained through the NIS database including age, sex, race, median income quartiles, and insurance status. To further classify patients, comorbidities were also abstracted including history of obesity, alcohol use, smoking, asthma, chronic bronchitis, obstructive sleep apnea, diabetes mellitus without coma, hypertension, and hypothyroidism using ICD-9 codes (Table 1). Patients were assessed for a co-diagnosis of esophageal reflux. Patients with reflux esophagitis were not included in this study in order to use the co-diagnosis (Diagnoses 4-12) of GERD without esophagitis as a proxy for adequately treated disease [9-11].

Outcomes

The primary outcomes of this study were in-hospital morbidity and mortality. Morbidities from PH that were studied included pulmonary hemorrhage, pulmonary embolism, and right heart failure/cor pulmonale. Major adverse cardiovascular events (MACE), defined by stroke, acute myocardial infarction, and cardiac arrests were also identified. Secondary outcomes included common hospital complications including urinary tract infection, aspiration pneumonia, and acute renal failure were assessed. Finally, hospital length of stay and hospital charges were also analyzed between patients with and without GERD. 

Hospital charges

Hospital charges were adjusted to account for inflation. The mean charges were reported as raw charges and then adjusted to the December 31, 2013, equivalent rate using the U.S. Bureau of Labor Statistics Inflation Calculator (https://www.bls.gov/data/inflation_calculator.htm).

Statistical analysis

Through the discharge weight provided in the NIS database, the national estimates were created, as previously utilized by HCUP methods. We used chi-squared analysis to compare the categorical comorbidity and socio-demographic variables to identify which variables to include in the binomial logistic regression. A separate binomial logistic regression was utilized to analyze the complications - specifically, the morbidity and mortality associated with PH, stratifying for patients with a diagnosis of gastro-esophageal reflux. All covariates that were identified to be significantly associated with mortality were included in the multivariable models. All statistical analysis was completed using SPSS version 27 (IBM Corp., Armonk, NY).

## Results

Characteristics of PH-related hospitalizations

We identified 159,394 patients with a primary diagnosis of PH which corresponds with approximately 800,000 admissions nationally. Of these patients, 14.3% had an associated diagnosis of esophageal reflux. The predominant age group was 61- to 79-year-olds. Most of the patients (61.6%) were Caucasian and female (66.0%), - a gender discrepancy that has been previously reported [12]. A majority of patients (59.6%) were insured under Medicare, the second most common payor being private insurance (23.3%). Of all the comorbidities, the only nonsignificant difference between the groups was the history of smoking, the rest were significantly different (Tables 2-3).

**Table 2:** Patient characteristics data (N) *Significance level p<0.05

Variable	No GERD diagnosis	GERD diagnosis	P-value
Age			< .001*
19 to 29	5062 (83%)	1062 (17%)	
30 to 50	24672 (88%)	3511 (12%)	
51 to 60	20275 (85%)	3558 (15%)	
61 to 79	53660 (87%)	7744 (13%)	
≥ 80	34753 (87%)	5097 (13%)	
Race			< .001*
Caucasian	78226 (84%)	15029 (16%)	
African American	23997 (77%)	6990 (23%)	
Hispanic	13750 (72%)	5255 (28%)	
Asian, Pacific Islander, Native American	11112 (69%)	5035 (31%)	
Median income quartiles			< .001*
0-25^th^ Percentile	30822 (87%)	4747 (13%)	
26-50^th^ Percentile	34328 (88%)	4784 (12%)	
51-75^th^ Percentile	33949 (88%)	4617 (12%)	
76-100^th^ Percentile			
Sex			< .001*
Males	48078 (91%)	4723 (9%)	
Females	93378 (88%)	13215 (12%)	
Insurance cohorts			< .001*
Private insurance	33021 (89%)	4103 (11%)	
Medicaid	17389 (91%)	1720 (9%)	
Medicare	84220 (88%)	11538 (12%)	
No insurance	6792 (92%)	611 (8%)	
Obesity			< .001*
Not obese	126169 (89%)	15427 (11%)	
Obese	15350 (86%)	2448 (14%)	
History of alcohol use			< .001*
No alcohol use	138389 (89%)	17599 (11%)	
Alcohol use	3130 (92%)	276 (8%)	
History of smoking			.750
No smoking	130210 (89%)	16460 (11%)	
Smoking	11308 (89%)	1416 (11%)	
Asthma			< .001*
No asthma	130568 (89%)	15617 (11%)	
Asthma	10861 (83%)	2258 (17%)	
Chronic bronchitis			< .001*
No bronchitis	107856 (89%)	13377 (11%)	
Chronic bronchitis	33663 (88%)	4498 (12%)	
Obstructive sleep apnea			< .001*
No OSA	134765 (89%)	16400 (11%)	
OSA	6754 (82%)	1475 (18%)	
Non-coma diabetes mellitus			< .001*
No DM	104670 (89%)	13372 (11%)	
DM	36849 (89%)	4503 (11%)	
Hypertension			< .001*
No HTN	97305 (90%)	11134 (10%)	
HTN	44213 (87%)	6742 (13%)	
Hypothyroidism			< .001*
No hypothyroidism	125452 (89%)	14753 (11%)	
Hypothyroidism	16067 (84%)	3122 (16%)	

**Table 3:** Predictors of gastroesophageal reflux disease (GERD) based on socio-demographics and co-morbidities *Significance level p<0.05

Variable	P-value	Odds ratio (95% CI)
Age		
19 to 29	Reference category	
30 to 50	< .001*	1.60 (1.38-1.85)
51 to 60	< .001*	1.91 (1.65-2.22)
61 to 79	< .001*	1.61 (1.39-1.86)
≥ 80	< .001*	1.49 (1.28-1.73)
Race		
Caucasian	Reference category	
African American	< .001*	0.91 (0.87-0.96)
Hispanic	< .001*	0.67 (0.62-0.73)
Asian, Pacific Islander, Native American	< .001*	0.69 (0.62-0.76)
Gender		
Males	Reference category	
Females	< .001*	1.37 (1.31-1.42)
Insurance status		
Private insurance	Reference category	
Medicaid	< .001*	0.77 (0.72-0.84)
Medicare	< .001*	1.12 (1.06-1.18)
No insurance	< .001*	0.62 (0.54-0.72)
Median income quartiles		
0-25^th^ percentile	Reference category	
26-50^th^ percentile	.018^*^	0.94 (0.89-0.99)
51-75^th^ percentile	< .001*	0.84 (0.80-0.90)
76-100^th^ percentile	< .001*	0.85 (0.80-0.89)
Obesity		
Not obese	Reference category	
Obese	.006^*^	1.08 (1.02-1.15)
History of alcohol use		
No alcohol use	Reference category	
Alcohol use	.028^*^	0.86 (0.75-0.98)
History of smoking		
No smoking	Reference category	
Smoking	.919	1.00 (0.94-1.07)
Asthma		
No asthma	Reference category	
Asthma	< .001*	1.53 (1.45-1.63)
Chronic bronchitis		
No bronchitis	Reference category	
Chronic bronchitis	< .001*	1.10 (1.06-1.15)
Obstructive sleep apnea		
No OSA	Reference category	
OSA	< .001*	1.46 (1.37-1.57)
Non-coma diabetes mellitus		
No DM	Reference category	
DM	< .001*	0.90 (0.87-0.94)
Hypertension		
No HTN	Reference category	
HTN	< .001*	1.32 (1.28-1.38)
Hypothyroidism		
No hypothyroidism	Reference category	
Hypothyroidism	< .001*	1.49 (1.42-1.56)

Prediction of GERD with respect to socio-demographics and comorbidities

Compared to the 18- to 30-year-old cohort, patients in all other age groups had higher odds of having GERD, with the 51- to 60-year-olds having the highest odds (OR 1.91). Compared to Caucasians, all other races (including African Americans, Hispanics, and the Asian, Pacific Islander, and Native American groups) had decreased odds of having GERD. Patients with GERD were more likely to be female (OR 1.37) and less likely to be from the higher compared to the lowest 25% median income quartile. Patients with Medicaid and those without insurance were less likely to have GERD compared to those patients with private insurance (OR 0.77 and 0.62, respectively). This was in contrast to those patients who had Medicare, who were more likely (OR 1.12) to have GERD compared to those with private insurance (Table 2).

**Table 4:** Patient morbidity and mortality data (n) *Significant level p<0.05

Variable	No GERD Diagnosis	GERD Diagnosis	P-Value
Urinary tract infection			< .001*
No UTI	131920 (89%)	16827 (11%)	
UTI	9599 (90%)	1048 (10%)	
Acute renal failure			< .001*
No ARF	131625 (89%)	16951 (11%)	
ARF	9894 (91%)	925 (9%)	
Myocardial infarction			< .001*
No myocardial infarction	134608 (89%)	17303 (11%)	
Myocardial infarction	6911 (92%)	572 (8%)	
Aspiration pneumonia			< .001*
No aspiration PNA	140413 (89%)	17655 (11%)	
Aspiration PNA	1106 (83%)	220 (17%)	
Cardiac testing			< .001*
No cardiac testing	116802 (88%)	15260 (12%)	
Cardiac testing	24717 (90%)	2615 (10%)	
Pulmonary hemorrhage			< .007*
No hemorrhage	140320 (89%)	17758 (11%)	
Hemorrhage	1199 (91%)	117 (9%)	
Pulmonary embolism			< .001*
No PE	138060 (89%)	17511 (11%)	
PE	3459 (90%)	364 (10%)	
Right heart failure			< .001*
No RHF	139359 (89%)	17633 (11%)	
RHF	2160 (90%)	242 (10%)	
Stroke			.820
No stroke	140135 (89%)	17697 (11%)	
Stroke	1384 (89%)	178 (11%)	
Cardiac arrest			< .001*
No cardiac arrest	140495 (89%)	17808 (11%)	
Cardiac arrest	1023 (94%)	68 (6%)	
Acute HF exacerbation			< .001*
No exacerbation	134771 (89%)	16828 (11%)	
HF exacerbation	6747 (87%)	1048 (13%)	
Respiratory failure			< .001*
No resp failure	134445 (89%)	17257 (11%)	
Resp failure	7074 (92%)	618 (8%)	
Mortality			< .001*
Dead	135281 (89%)	17324 (11%)	
Alive	6170 (91%)	619 (9%)	

In logistic regression analyses, patients with PH who were diagnosed with GERD were more likely to be obese (OR 1.08), have asthma (1.53), chronic bronchitis including COPD (OR 1.10), obstructive sleep apnea (OR 1.46), hypertension (OR 1.32) and hypothyroidism (OR 1.49). Patients with GERD were less likely to have a history of alcohol use (OR 0.86) and were less likely to have diabetes (OR 0.90). There was no significant association with a history of smoking (Table 2).

Prediction of PH complications in GERD patient group

**Figure 1:**
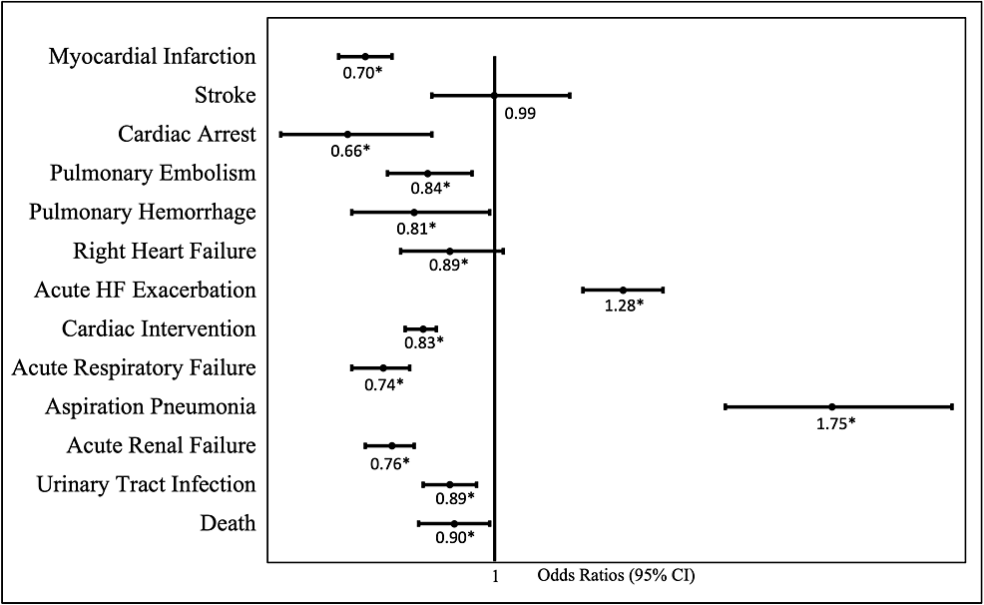
Odds ratio of complications in patients with diagnosed gastroesophageal reflux disease (GERD) This figure demonstrates the odds ratio of complications occurring in patients that have been diagnosed with GERD with a 95% confidence interval.

PH patients who had a myocardial infarction were 30% less likely to also have a diagnosis of GERD. Patients that had an ischemic stroke were, insignificantly, 1% less likely to have GERD. Patients who had a cardiac arrest were 34% less likely to also have GERD. Complications of PH included an increased risk for pulmonary embolisms and pulmonary hemorrhage. PH patients with GERD were 16% less likely to also have a pulmonary embolism, while they were 19% less likely to have a pulmonary hemorrhage. Patients with right heart failure were 11% less likely to also have GERD. Interestingly, patients that underwent cardiac catheterization with or without coronary angiography were 17% less likely to also have GERD. Patients with GERD, however, did have 28% increased odds of having a heart failure exacerbation (Figure 1).

PH patients that had acute respiratory failure were 26% less likely to have GERD. In addition, those that developed urinary tract infections or acute renal failure were also 11% and 24% less likely to also be diagnosed with GERD. However, not surprisingly, PH patients did have a 75% increased risk of aspiration pneumonia compared to those without GERD. Overall mortality rate in the PH population without GERD was 4.56% compared to the rate of 3.57% in PH patients diagnosed with GERD (Figure 1).

Healthcare utilization trends

**Figure 2:**
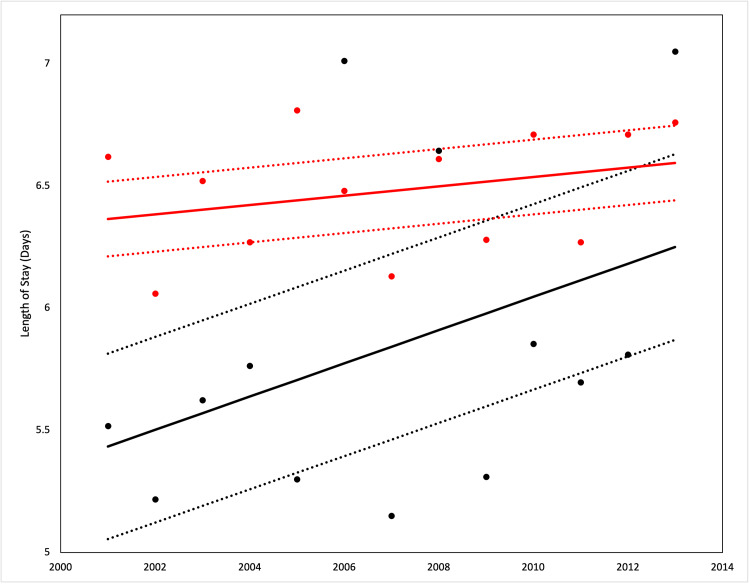
Length of stay between study population and those with gastroesophageal reflux disease (GERD) This figure demonstrates the difference in length of stay of patients with GERD (black line) compared to the overall population (red line). The dashed lines represent a 95% confidence interval.

**Figure 3:**
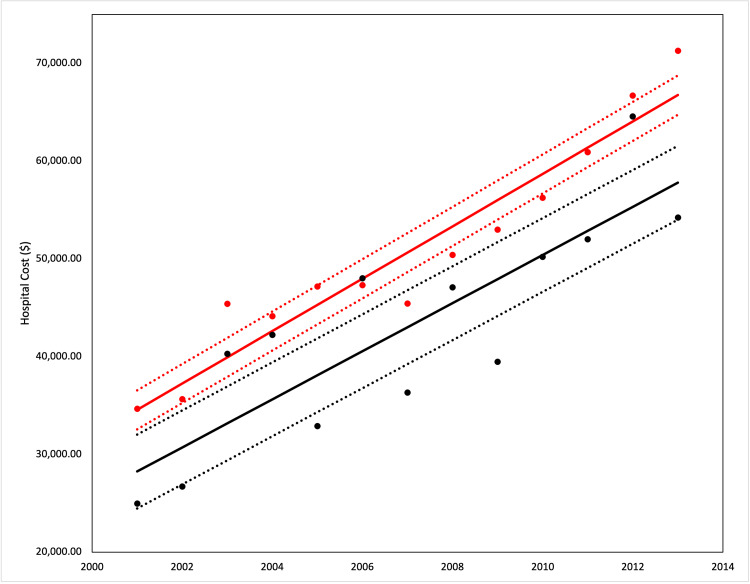
Hospital costs between study population and those with gastroesophageal reflux disease (GERD) This figure demonstrates the difference in hospital charges of patients with GERD (black line) compared to the overall population (red line). The dashed lines represent a 95% confidence interval.

The average LOS for the population of patients with PH admitted from 2001 to 2013 increased from 6.62 days to 6.76 days. Patients who had a co-diagnosis of GERD had an average LOS that trended from 5.5 days to 7.0 days but had significantly lower LOS compared to those without GERD. Generally, LOS increased significantly in both patient groups from 2001 to 2013. The average hospital costs for patients with PH increased from $35,000 in 2001 to $71,000 in 2013. PH patients who were diagnosed with GERD had an average hospital cost of $28,009 in 2001 up to $58,000 in 2013. All hospital costs were adjusted for inflation. Both LOS and hospital costs differed between the patients with and without GERD and were statistically significant. PH patients with GERD had significantly lower costs than those without GERD in from 2001 to 2013, although costs increased significantly in both patient groups from 2001 to 2013 (Figures 2-3).

## Discussion

PH is a rare disease that significantly affects a patient's lifestyle, mobility, and well-being [13]. In our study, we examined the relationship between PH and GERD without esophagitis. Through our investigation, we found that GERD without esophagitis in patients with PH was associated with decreased odds of myocardial infarction, cardiac arrest, pulmonary embolism, acute respiratory failure, and renal failure among comorbidities or complications. Our findings suggest that there may be a connection between GERD, PH, and many of the sequelae that arise from it. Further investigation may be warranted to explore the role of acid-reducing therapy in GERD patients with or at high risk for PH.

The distribution of comorbidities and sociodemographic factors associated with the general diagnosis of GERD is consistent with that reported in previous studies [14]. We found that 18-30-year-old PH patients had lower odds of having GERD compared to the general PH population, with higher ORs in the 30-50-year-old and 51-60 patients. These findings are similar to those reported in a recent study in 2018 examining GERD prevalence among patients in America [14]. In addition, in separate race and gender analyses, we similarly found a higher OR for GERD in Caucasians and females, showing a similar demographic distribution as the American public [15]. Thus, our patient database diagnosed with PH and GERD roughly models the American public diagnosed with GERD.

In addition to socio-demographics, we examined many of the serious complications of PH. PH not infrequently manifests as right ventricular diastolic and even left ventricular dysfunction, which can result in heart failure and chronic multiorgan damage [16]. Yet, our findings suggest that the diagnosis of GERD with adequate management may be protective in PH patients. GERD was associated with a decreased risk of multiple associated conditions, notably cardiac arrest, and myocardial infarction.

Our analysis of PH complications in the setting of diagnosed gastroesophageal reflux without esophagitis suggests a possible mechanism for the protective role of acid-reducing pharmacotherapy. Given that we examined only patients with reflux that did not have esophagitis, these patients were prescribed acid modulation therapy which has been shown to prevent esophageal inflammation [16]. Through the inhibition of H+ ATPase channels, PPIs prevent acidification of gastric contents that irritate the upper gastrointestinal mucosal lining. It has been previously shown that stomach contents are aspirated in small quantities at night in many patients, even in young and healthy adults [17]. This micro-aspiration has been linked to a variety of pulmonary pathology, including idiopathic pulmonary fibrosis [18]. If PPIs are able to reduce the acidity and inflammation in this aspirated content, it may slow the progression of PH. This disease retardation may in turn significantly reduce the odds of developing subsequent cardiac pathology. This hypothesis deserves to be tested prospectively.

The link between micro-aspirations, PPIs usage, and PH complications is further highlighted in acute hospital stays. We found that from 2001-2013, patients with GERD as a complication of PH had shorter hospital stays than the control group. We hypothesize that this difference may again be attributed to the use of PPIs. Debilitated or severely ill patients often have increased micro-aspirations leading to macro-aspirations and pneumonia, among other pathologies, thereby prolonging hospital stays [19]. By reducing the acidity of these aspirations, acid-modulating medications may protect patients with GERD from exacerbations of their PH and reduce their hospital stay. Thus, the diagnosis of GERD may counterintuitively have a protective role because of PPI treatment. Treatment of PH with PPIs alone or PPIs with domperidone (dopamine antagonist) should be considered in future studies.

In addition to the protective effects of GERD, we found other risk factors, including obesity, the treatment of which may result in improved morbidity and mortality. It has been previously shown that PH is associated with obesity [20,21]. Obesity is also known to be associated with an increased risk of GERD; studies have found the incidence of GERD in the United States to be 18.1%-27.8% with an OR of 1.58 of having GERD in obese people [22]. Patients with GERD and PH are likely to have increased adiposity. These factors may lead to increased chronic cardiac stress resulting in worsening heart failure. While the use of PPIs might have a cardioprotective role (as demonstrated by the decreased risk of myocardial infarction and cardiac arrest), obesity may worsen heart disease. This may explain that while our patients with GERD had a trend (nonsignificant) toward a decreased risk of acute heart failure exacerbation, their weight may have an inverse effect by worsening heart failure [23-25].

Limitations of our study include the difficulty in establishing a baseline severity of PH. We were not able to categorize patients based on the degree of PH severity. Another limitation is that our study included only adults and thus may not be applicable to adolescents and children. Finally, due to limitations in ICD-9 coding, the role of acid modulation therapy was unable to be directly tested. Future studies of the effect of GERD and its treatment on the severity of PH in various age groups may help elucidate underlying mechanisms and further our understanding of the role of GERD disease in PH.

## Conclusions

PH is a significant disease that affects patients on a daily basis. Looking at the relationship between GERD and PH, we found that the presence of GERD without active esophagitis was associated with decreased morbidity and mortality in patients with PH. We theorize there are protective mechanisms derived from acid-reducing pharmacotherapy, and that further retrospective studies controlling for the use of PPIs are required to establish a relationship between GERD and PH. It is of high importance to understand protective factors that prevent disease progression and morbidity/mortality from PH as the number of affected patients is on the rise. 

## References

[1] McLaughlin VV, Shillington A, Rich S (2002). Survival in primary pulmonary hypertension: the impact of epoprostenol therapy. Circulation.

[2] Hoeper MM, Ghofrani HA, Grünig E, Klose H, Olschewski H, Rosenkranz S (2017). Pulmonary hypertension. Dtsch Arztebl Int.

[3] Ramani G, Lam D, Park M (2014). Clinical significance of gastroesophageal reflux disease and proton pump inhibitor use in pulmonary arterial hypertension. J Heart Lung Transplant.

[4] Gaude GS (2009). Pulmonary manifestations of gastroesophageal reflux disease. Ann Thorac Med.

[5] Tutuian R, Castell DO (2006). Review article: complete gastro-oesophageal reflux monitoring - combined pH and impedance. Aliment Pharmacol Ther.

[6] Hoppo T, Jarido V, Pennathur A (2011). Antireflux surgery preserves lung function in patients with gastroesophageal reflux disease and end-stage lung disease before and after lung transplantation. Arch Surg.

[7] Ruffenach G, Hong J, Vaillancourt M, Medzikovic L, Eghbali M (2020). Pulmonary hypertension secondary to pulmonary fibrosis: clinical data, histopathology and molecular insights. Respir Res.

[8] Hossain A, Mittal A, Hossain S, Ahlawat S: (2021). Role of gastroesophageal reflux disease in patients admitted for a primary diagnosis of pulmonary hypertension. Am Thorac Soc.

[9] Gerson LB, McLaughlin T, Balu S, Jackson J, Lunacsek O (2012). Variation of health-care resource utilization according to GERD-associated complications. Dis Esophagus.

[10] Mak SM, Strickland N, Gopalan D (2017). Complications of pulmonary hypertension: a pictorial review. Br J Radiol.

[11] Luo Y, Jiang C, Krittanawong C (2019). Systemic sclerosis and the risk of perioperative major adverse cardiovascular events for inpatient non-cardiac surgery. Int J Rheum Dis.

[12] Peacock AJ, Murphy NF, McMurray JJ, Caballero L, Stewart S (2007). An epidemiological study of pulmonary arterial hypertension. Eur Respir J.

[13] Delcroix M, Howard L (2015). Pulmonary arterial hypertension: the burden of disease and impact on quality of life. Eur Respir Rev.

[14] Yamasaki T, Hemond C, Eisa M, Ganocy S, Fass R (2018). The changing epidemiology of gastroesophageal reflux disease: are patients getting younger?. J Neurogastroenterol Motil.

[15] Eusebi LH, Ratnakumaran R, Yuan Y, Solaymani-Dodaran M, Bazzoli F, Ford AC (2018). Global prevalence of, and risk factors for, gastro-oesophageal reflux symptoms: a meta-analysis. Gut.

[16] Mermelstein J, Mermelstein AC, Chait MM (2016). Proton pump inhibitors for the treatment of patients with erosive esophagitis and gastroesophageal reflux disease: current evidence and safety of dexlansoprazole. Clin Exp Gastroenterol.

[17] Gleeson K, Eggli DF, Maxwell SL (1997). Quantitative aspiration during sleep in normal subjects. Chest.

[18] Lee JS, Collard HR, Raghu G (2010). Does chronic microaspiration cause idiopathic pulmonary fibrosis?. Am J Med.

[19] Rouzé A, Jaillette E, Nseir S (2018). Relationship between microaspiration of gastric contents and ventilator-associated pneumonia. Ann Transl Med.

[20] Frank RC, Min J, Abdelghany M (2020). Obesity is associated with pulmonary hypertension and modifies outcomes. J Am Heart Assoc.

[21] Min J, Feng R, Badesch D (2020). Obesity in pulmonary arterial hypertension (PAH): the Pulmonary Hypertension Association Registry (PHAR). Ann Am Thorac Soc.

[22] Chang P, Friedenberg F (2014). Obesity and GERD. Gastroenterol Clin North Am.

[23] Chen Q, Zhuang H, Liu Y (2012). The association between obesity factor and esophageal caner. J Gastrointest Oncol.

[24] Souza RF (2016). From reflux esophagitis to esophageal adenocarcinoma. Dig Dis.

[25] Rosenkranz S, Howard LS, Gomberg-Maitland M, Hoeper MM (2020). Systemic consequences of pulmonary hypertension and right-sided heart failure. Circulation.

